# Extended bound states in the continuum in a one-dimensional grating implemented on a distributed Bragg reflector

**DOI:** 10.1515/nanoph-2021-0478

**Published:** 2021-11-23

**Authors:** Emilia Pruszyńska-Karbownik, Mikołaj Janczak, Tomasz Czyszanowski

**Affiliations:** Institute of Physics, Lodz University of Technology, ul. Wólczańska 217/221, 93-005, Łódź, Poland

**Keywords:** bound states in the continuum, distributed Bragg reflector, one dimensional grating, semiconductor microcavity

## Abstract

Bound states in the continuum (BICs) are observed in optical cavities composed of a high refractive index periodic structure embedded in significantly lower refractive index surroundings, enabling vertical confinement of the grating modes. Here, we propose a vertically nonsymmetric configuration, implemented on a high refractive index bulk substrate with a one-dimensional grating positioned on a distributed Bragg reflector (DBR). In this configuration, the grating modes are leaky, which could prohibit the creation of a BIC if the grating was implemented on uniform substrate. However, the judiciously designed DBR on which the grating is implemented reflects nonzero diffraction orders induced by the grating. We found that the laterally antisymmetric optical modes located at the center of the Brillouin zone of this structure create BICs that are robust against changes in the grating parameters, as long as the DBR reflects the diffraction orders. The configuration enables a high degree of design freedom, facilitating the realization of very high quality factor cavities in conventional all-semiconductor technology.

## 1 Introduction

Bound states in the continuum (BICs) were predicted on the basis of quantum mechanics nearly a century ago by von Neuman and Wigner [1]. However, they have received most attention in the field of optics, beginning with [2], as they enable electromagnetic wave confinement to subwavelength dimensions with an infinite quality (*Q*) factor. BICs are nonradiating electromagnetic resonant states localized in open photonic systems (above the light line), although they can coexist with a continuous spectrum of unbound states [3, 4]. Two types of BICs can be formed in the center of Brillouin zone (Γ point) of periodic structures. The first type is a symmetry protected (SP) BIC that appears when the symmetry of a grating mode prohibits its coupling to a symmetric plane wave propagating perpendicularly to the grating surface. Such modes are antisymmetric with respect to at least some of the vertical symmetry planes or axes of the grating. In general, the subwavelength periodic structure admits a single leaky channel related to zero diffraction order emission, which is absent in the case of antisymmetric modes, and therefore any radiation is prohibited [5]. SP BICs do not disappear when the parameters of the grating vary, as long as the structure is subwavelength. The second type of BIC may arise when a mode is symmetric with respect to the grating symmetry or the grating is not subwavelength. Emission of the mode occurs by a single channel, for example by a single diffraction order. By careful tuning of the grating parameters, destructive interference can prohibit emission in such a configuration [6, 7], creating a so-called resonance-trapped BIC. A particular type of resonance-trapped BIC is the Friedrich–Wintgen BIC [7]. This BIC is induced when the dispersion curves of strongly coupled nonorthogonal resonances approach each other. Avoided crossing with noticeable separation of the frequencies then appears [8] and leads to an increase in the *Q*-factor of one of the modes. This mechanism can be observed not only in periodic configurations, but also in finite single resonators where dispersion curves of resonances of various origins approach each other [9, 10].

Real nonideal implementation of BIC-based cavities inevitably leads to the collapse of the infinite *Q*-factor, because of technological imperfections, roughness, internal absorption, and the finite size of the structure. However, BIC-based cavities are still very useful for many optical and photonic applications requiring high *Q*-factors [11, 12]. These cavities share a common feature, which is a low refractive index substrate or membrane configuration. High refractive index contrast between the periodic structure and the surroundings is required to support the waveguide mechanism in the periodic structure. Such designs make it difficult to fabricate electrically driven devices, since the air or low refractive index materials (typically dielectrics) are nonconducting. In comparison, all-semiconductor structures such as, for example, periodic structures implemented on a bulk substrate, offer important advantages. They are robust and immune to mechanical failure, and have the potential to be used in electrically driven devices. All-semiconductor high *Q*-factor cavities based on BICs could open new possibilities in diverse applications and research fields, such as low-threshold nano lasers [13], single photon sources [14], Bose–Einstein condensation [15, 16], superfluidity [17], Raman scattering [18], quantum optics [19], electrooptics [20], and the quantum Hall effect [21].

However, all-semiconductor structures inevitably have low refractive index contrast between the substrate and the periodic structure, which hinders the formation of a BIC. In the case of SP BICs, the presence of the substrate modifies the subwavelength condition for light propagating towards the substrate, as the period of the structure must satisfy the relation *L*/*λ* < *n*_s_^−1^, *n*_a_^−1^ where *n*_s_ and *n*_a_ are the refractive indices of the substrate and superstrate, respectively. Typically, the superstrate is air and substrates can be composed of materials such as Si, GaAs, GaSb, Ge, or InP, with refractive indices larger than three in infrared. The creation of SP BIC in configurations with such high substrate refractive indices requires a periodic structure with a small lattice constant, which is technologically demanding to fabricate. The creation of BICs additionally requires the waveguiding condition to be satisfied by the periodic structure, which according to waveguide theory directly implies that the effective refractive index [22] of the mode is larger than *n*_s_ and *n*_a_ [23]. When the refractive index contrast between periodic structure and substrate is low, this condition is difficult to meet [24], since the refractive index of the periodic structure is effectively lowered by air infiltrating the structure. An interesting approach to a vertically nonsymmetric configuration is the design demonstrated in [8], in which the metal grating reflects the light from the substrate side and the glass cavity confines the light. However, the presence of metal introduces loss that prevents the creation of a true BIC.

In this paper, based on the example of a one-dimensional grating implemented on a distributed Bragg reflector (DBR) with air above the grating, we numerically demonstrate a BIC that is extended in the grating parameters space, positioned in the range *L*/*λ* < *n*_a_^−1^ that is independent on the refractive index of the substrate (*n*_s_). We show that in this configuration radiative losses of laterally antisymmetric modes can be entirely eliminated by judicious design of the grating and DBR. Although the grating itself does not provide a waveguiding mechanism, the presence of the DBR eliminates any leakage of the light that propagates toward the substrate from the grating. As a result, a BIC is formed that is robust against changes in the grating parameters as long as the DBR reflects the diffraction orders of the grating and the mode is formed in the center of Brillouin zone of the periodic structure. When this configuration is entirely composed of semiconductors, as exemplified in Supplementary materials, the epitaxial composition of the layers constitutes a half-configuration of a vertical-cavity surface-emitting laser (VCSEL) that can be electrically driven. Since this configuration does not require a waveguiding mechanism in the grating layer, unlike vertically nonsymmetric configurations without a DBR supporting BIC [24], large variations in the heights and fill factors enabling BIC are possible. This significantly relaxes the technological requirements.

In Section 2, we present the details of the configuration considered in this work and the numerical method used for its analysis. In Section 3, we present the mechanism of BIC formation. In Section 4, we demonstrate the properties of the BIC. In Section 5, we present the dependence of the *Q*-factor on the number of grating stripes. This example demonstrates the possibility of achieving a very high *Q*-factor in a structure with a finite number of grating stripes.

## 2 Structure and numerical method

**Figure 1:**
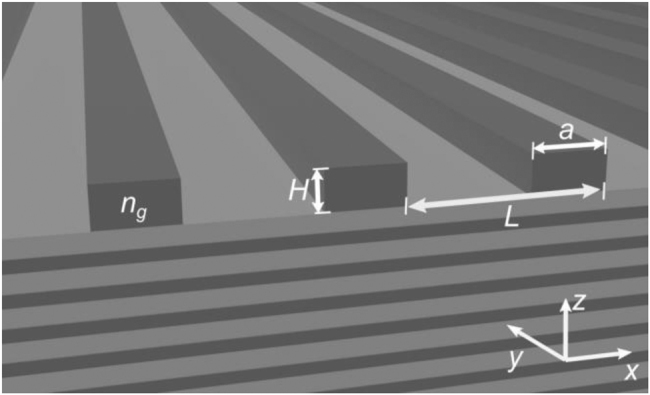
Schematic cross-section of a grating implemented on a DBR. The geometrical parameters of the grating and coordinate system are defined graphically. The refractive index of the grating and the larger refractive index of the DBR (dark grey) are both *n*_H_ = 3.52; the lower refractive index (light grey) is *n*_L_ = 2.2.

The exemplary structure analyzed here is composed of a one-dimensional grating, periodic in the *x* direction and translationally invariant in the *y* direction. The structure is not symmetric along the *z* axis, as the grating is positioned on a 35-pair DBR with a semi-infinite substrate and semi-infinite air superstrate. The refractive index of the grating and substrate is *n*_H_ = 3.52, that of air is *n*_a_ = 1 and the DBR is composed of layers with refractive indices of *n*_L_ = 2.2 and *n*_H_ = 3.52 (Figure 1). The refractive indices can correspond, for example, to the refractive indices of SiN_*x*_ (*n*_L_) and amorphous Si (*n*_H_) in the near infrared. In Supplementary material, as an example of the versatility of our approach, a configuration is shown of which the refractive indices correspond to arsenide-based materials with significantly lower refractive index contrast (*n*_L_ = 2.95 and *n*_H_ = 3.52). The geometrical parameters of the structure are shown in Figure 1, including the grating period (*L*), the height of the grating stripes (*H*), and the grating duty cycle (*F*). The grating duty cycle is defined as the ratio of the grating stripe width (*a*) to the period (*L*), which is *F* = 0.35 in the considered configuration. The thicknesses of the DBR layers are designed to reflect first and second diffraction orders emitted by the grating, as will be discussed further. Details of the structure are collected in Table 1. However, particular parameters of the structure can be modified, which will be indicated in the figures presented in this paper. The values of other parameters, shown in Table 1, remain constant. The dimensions of the grating are inspired by the parameters of a real-world realization of a subwavelength grating described in [25], to indicate the feasibility of a high quality realization of the proposed configuration. Particularly small values for *F* and *H* enable facile grating realization by electron beam lithography for research applications or by nanoimprint lithography [26] on a mass production scale. We study the modes supported by this structure in transverse electric (TE) polarization, i.e., with an electric field polarized along the stripes in the *y* direction and antisymmetric with respect to the grating in the *x* direction, which prohibits the zeroth diffraction order [5].

**Table 1:** Geometrical details of the structure for unity wavelength.

Layer	Dimensions	Value	Refractive index
Grating	Height *H*	0.191	3.52
	Period *L*	0.950	
	Fill-factor *F*	0.350	
Low-refractive-index DBR layer	Thickness	0.137	2.2
High-refractive-index DBR layer	Thickness	0.086	3.52

We use the plane wave admittance method [27] to calculate the eigenvalues and electromagnetic fields of the modes. The computed electromagnetic wave along the *x* direction is in the form given by Bloch’s theorem:

(1) $$\Psi \left(x\right)=e^{ik_{x}x}f\left(x\right)$$

where *f*(*x*) is a periodic function with the same period *L* as the grating period and *k*_*x*_ is an arbitrarily chosen number, ranging from –*π*/*L* to *π*/*L*. The eigenmodes of the structure are characterized by *k*_*x*_ and a resonance frequency ω that relates to the free space resonant wavelength *λ* = 2*π*/*k*_0_, where *k*_0_ is a vacuum wavenumber. We consider modes located above the light line in the dissipative regime, which corresponds to *k*_*x*_ < *k*_0_, hence their resonant wavelengths are complex values with a nonzero imaginary part. The *Q*-factor of the modes is defined as *Q* = −0.5 Re(*λ*)/Im(*λ*). More details on the model can be found in Supplementary S1.

## 3 Mechanism of BIC formation

First, we start with a simple *Gedanken experiment* concerning the reduced structure described in Table 1, in which the DBR is replaced with a uniform layer of the averaged refractive index of the DBR, which is 2.89. All other parameters are unmodified. The averaged refractive index value of the grating given by the formula *n*_av_ = *F n*_H_ + (1 − *F*) *n*_a_ is 1.88, which is significantly lower than the averaged refractive index of the DBR (2.89) and is also lower than both of the refractive indices of the layers forming the DBR (*n*_H_ = 3.52, *n*_L_ = 2.2). Therefore, all the modes in this structure are leaky. The grating itself does not act as a waveguide, which would be necessary for the formation of a BIC in the grating [28]. The grating implemented on the DBR therefore acts only as a diffracting element.

**Figure 2:**
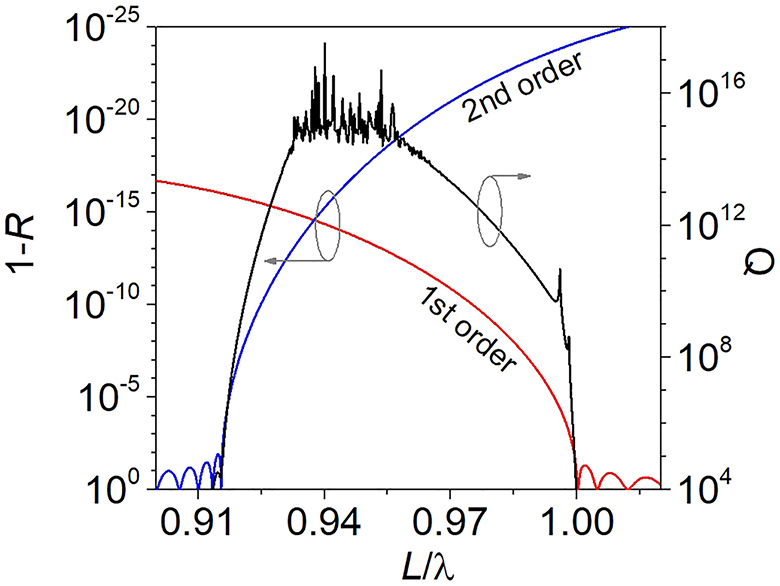
*Q*-factor (black line) of the mode indicated by (1) in Figure 2 and power reflectance of the DBR for the first (red line) and second (blue line) diffraction orders as a function of *L*/*λ*.

The normal incidence planewave illumination of the grating deposited on the DBR induces a zeroth diffraction order towards air and up to a second diffraction order that propagate towards the DBR. The propagation directions of the first and second diffraction orders are governed by the grating equation [29]. The DBR is designed to reflect the vertical components of wavevectors related to the first (red line) and second (blue line) diffraction orders of the grating, as Figure 2 illustrates in the spectral range 0.9 < *L*/*λ* < 1. When concerning laterally antisymmetric mode residing within the grating, located at the center of the Brillouin zone at Γ point (*k*_*x*_ = 0), the structure prevents any emission of the light since zeroth order emission is prohibited by the mode antisymmetry [5].

**Figure 3:**
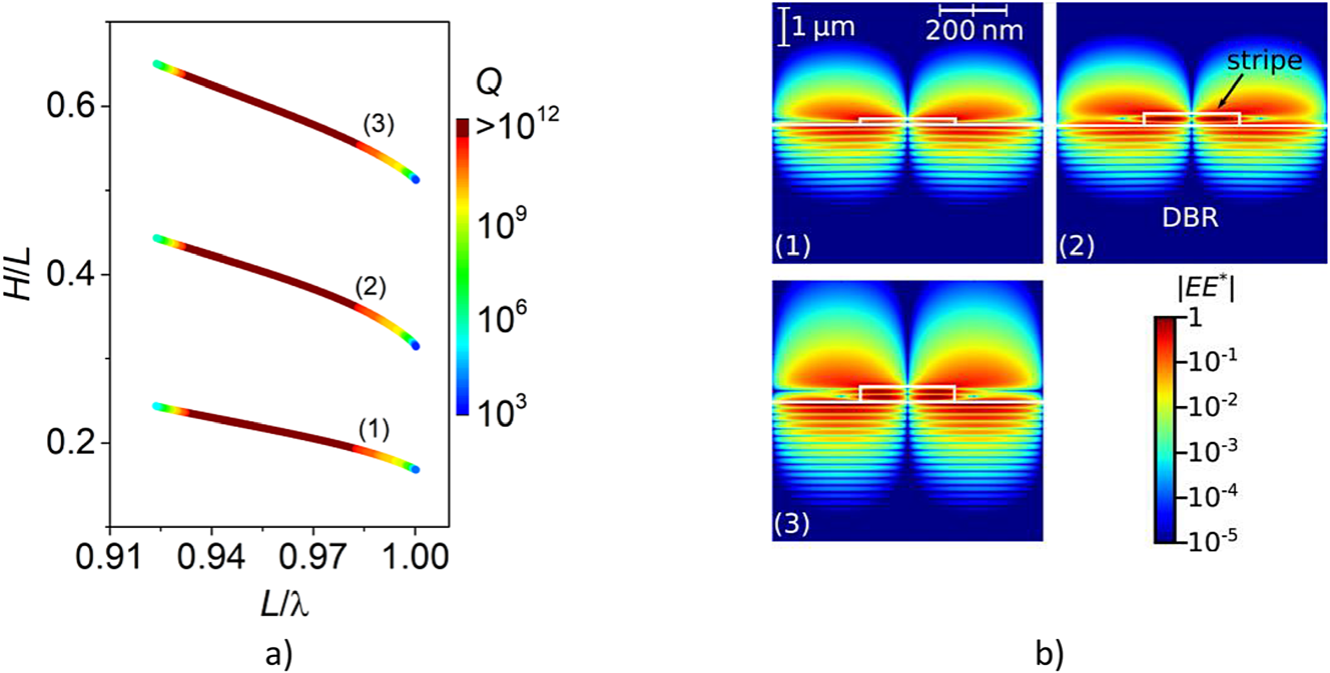
*Q*-factors of the antisymmetric modes indicated by colors at the Γ-point of the Brillouin zone in the domain of normalized stripe thickness *H* and wavelength λ in a) and light intensity distribution in b) for the modes indicated by numbers in a). A single period is represented in the figures. The edges of the grating stripe and the DBR are shown as white lines.

Figure 3a illustrates the dispersion curves of the antisymmetric modes in the configuration composed of the grating and the DBR. The dark red regions in the dispersion curves of the modes indicated by (1)–(3) in Figure 3a represent values above 10^12^. These values exceed the available computational precision and are therefore considered to be infinite, indicating BICs [5, 30] that do not disappear when the parameters of the grating vary in a limited range. The *Q*-factors of the modes remain infinite within an *L*/*λ* interval of 0.05 width and decay abruptly outside the dark red region, significantly below 1000, becoming untraceable by the numerical model. Therefore, these BICs can be considered as extended in the space of the wavelength and the grating parameters have similar properties to an SP BIC. The distributions of the modes indicated in Figure 3a are depicted in Figure 3b.

The range of the infinite *Q*-factor is constrained by the long and short wavelength limits of the DBR plateau for first and second diffraction orders (see Figure 2). The fluctuations of the *Q*-factor (black line in Figure 2) for *L*/*λ* < 0.92 correspond very well to the fluctuations of the DBR reflectivity spectrum for the second diffraction order. The short wavelength limit of the BIC coincides with the end of the reflection plateau for the first diffraction order and with the end of the subwavelength regime for emission towards air. Such a configuration positions the BIC very close to the diffraction limit (*L* = *λ*), which greatly relaxes the requirements on the fabrication in comparison to gratings supporting an SP BIC implemented on a uniform substrate [24]. The fluctuations of the *Q*-factor in the range 0.92 < *L*/*λ* < 0.97 result from limited numerical accuracy for values tending towards infinity.

The formation of a BIC in this configuration can be explained as follows. The DBR inhibits the propagation of the light in the *z* direction toward the substrate, inducing a condition similar to mirror symmetry. Therefore, the waveguide should be considered as the grating, together with the portion of the DBR in which the optical field resides. According to this interpretation, the effective refractive index of the waveguide should therefore be averaged over the grating and the portion of the DBR in which modes reside enabling waveguiding phenomenon that agrees with principles of BICs formation [5]. As the grating is not necessary to provide the waveguiding mechanism, BICs can be observed in a vast variety of structures and compositions. In Supplementary materials S2, geometrical details are given of an exemplary configuration with a GaAs grating and arsenide-based DBRs (*n*_L_ = 2.95 and *n*_H_ = 3.52) with a *Q*-factor above 10^11^. The light intensity distribution in this configuration is illustrated in Fig. S1.

## 4 Properties of BIC

**Figure 4:**
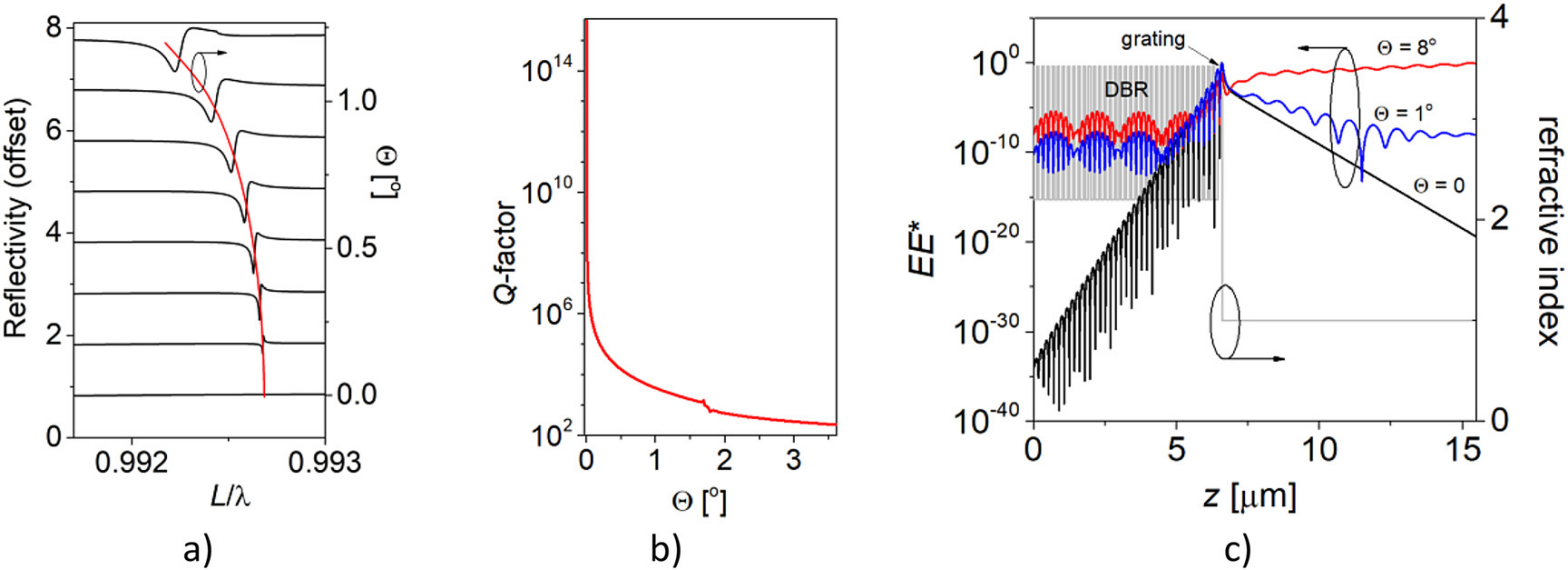
Reflection spectra at different incidence angles shown by black curves in a). The red curve represents the resonant wavelength of the mode indicated as (1) in Figure 3. *Q*-factor of mode (1) as a function of the transversal component of the wavevector *k*_x_ expressed by Θ (sinΘ = *k*_x_/*k*_0_) in b). Light intensity profiles along the z axis for various transversal components of the wavevector *k*_x_ expressed by Θ and different colors in c). The grey line represents the profile of the refractive index along the z axis.

Figure 4a illustrates the reflection spectra of the structure for different angles (Θ) of light incidence from the air side. The spectra show an abrupt transition from total reflection to total transmission at Θ ≠ 0, which is a fingerprint of a dissipative mode induced by Fano resonance [28]. The modification of Θ from 0 transforms the *Q*-factor abruptly from more than 10^12^ at the Γ point to below 10^3^ out of the Γ point (Figure 4b), as predicted by the theory of resonances in the near-BIC regime [30]. Figure 4c depicts the light intensity profiles for the structure along the vertical axis, which confirm the exponential decay of light for Θ = 0 in air and the DBR. Any deviation from Θ = 0 admits light propagation into air. This is crucial for enabling strong light–matter interaction, which allows excitation of the modes by light incident from the air side.

## 5 Finite grating structure

**Figure 5:**
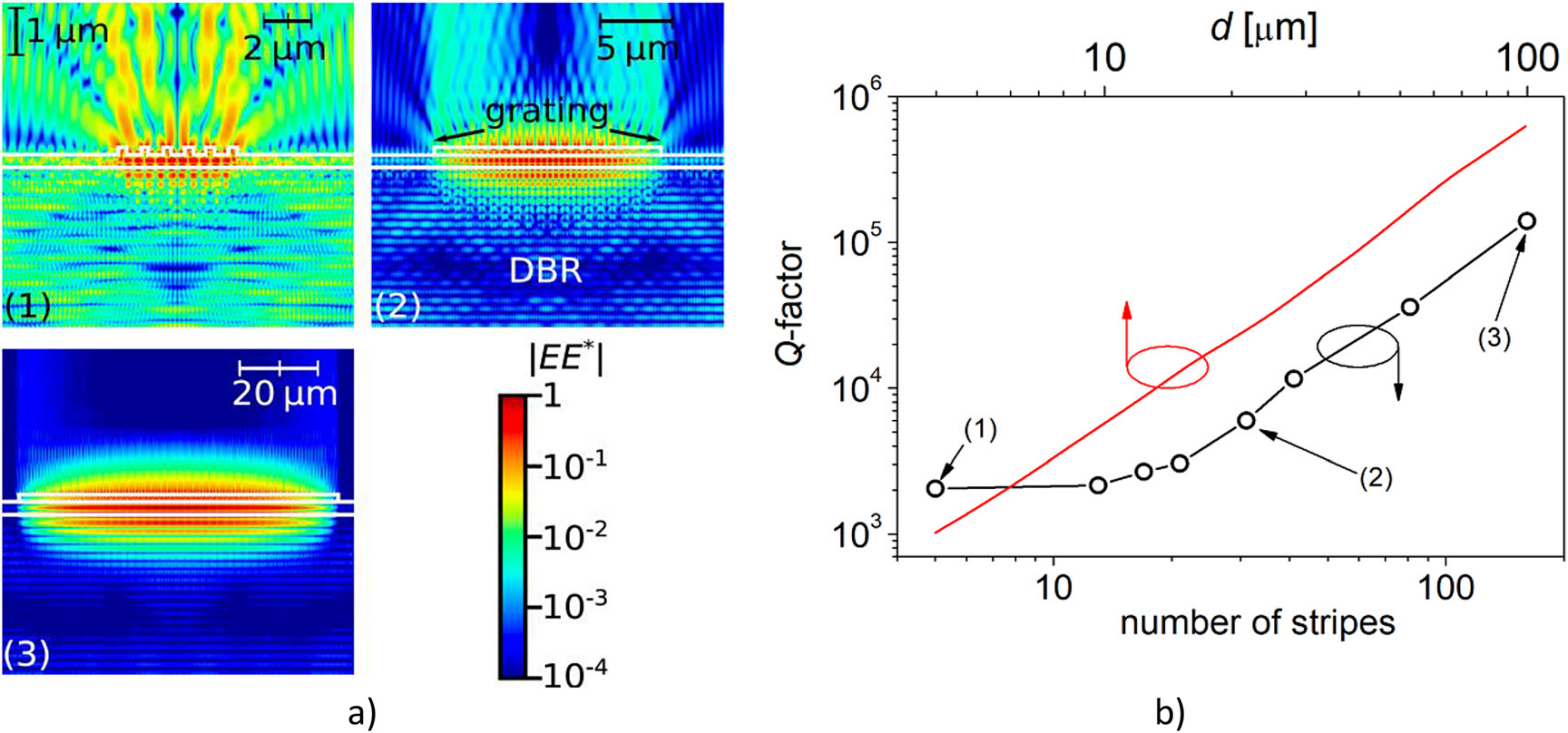
Light intensity distributions in the *x–z* plane on a logarithmic scale of colors for structures composed of 6, 20, and 100 grating stripes, indicated by numbers (1)–(3), respectively in a). *Q*-factor calculated in the *x–z* plane (black line and points) versus the number of grating stripes (bottom horizontal axis) and *Q*-factor calculated in the *y–z* plane (red line) versus length (*d*) of the grating stripes (top horizontal axis) in b). Numbers indicate the *Q*-factors of the structures presented in a).

The BIC analyzed here is perfectly decoupled from external waves by symmetry protection from the air side at normal incidence and by DBR reflection from the other side, resulting in an infinite *Q-*factor (see Figure 4b) and a vanishing resonance linewidth (see Figure 4a). Such properties are impractical; therefore, for most photonic applications it is necessary to tune the *Q*-factor to enable resonant coupling with external waves. In real-world implementations, an infinite *Q*-factor is unattainable, due to fabrication inaccuracy and the finite size of devices. Intentional tuning of the *Q*-factor is possible by fine-tuning the grating parameters, although this requires very high-precision grating fabrication. Changing the number of grating stripes enables more precise control of the *Q*-factor. In what follows, we perform a numerical analysis of a grating composed of stripes that are infinite in length. Absorbing boundary conditions are imposed on the lateral boarders of the computational window. In such a configuration, lateral optical losses in *y* direction are taken into account. Figure 5a illustrates the light intensity distribution in the *x*–*z* plane of the laterally fundamental mode in structures with various numbers of grating stripes. The configuration with a small number of stripes reveals substantial emission in the lateral direction. Increasing the number of stripes reduces the lateral emission and increases the *Q*-factor of the fundamental mode, as shown by the black line in Figure 5b. The *Q*-factors of the fundamental mode increase nearly exponentially with increasing numbers of grating stripes. The calculations for finite structures were limited to the maximal size of a structure composed of 100 stripes. This limitation was due to computational capability. To account for optical losses in the direction along the stripes (*y* direction), we performed three-dimensional calculations for an infinite number of grating stripes of various lengths. In this configuration, absorbing boundary conditions are imposed on the borders of the computational window in the *y* direction, whereas periodic boundary conditions are imposed for the *x* direction. In this configuration, the optical losses in the *x* direction are not taken into account. Exemplary light distributions of the fundamental mode in the *y*–*z* plain are presented in Fig. S2 in Supplementary materials. The red line in Figure 5b illustrates the *Q*-factor versus length of the grating stripes (*d*) for the fundamental mode. The curve is composed of a large number of calculated points, which are therefore not represented in the graph. Both the top and bottom horizontal axes of Figure 5b are linked in such a way that points on the black and red curves positioned on the same vertical line represent the *Q*-factors of structures with the same lateral dimensions. As can be seen, the *Q*-factor calculated in the *y–z* plane (*Q*_*y–z*_) is tenfold higher than that on the *x–z* plane (*Q*_*x-z*_). The *Q*-factor of the three-dimensional configuration (*Q*_3D_) can be approximated using the relation [31].

(2) $$Q_{3\text{D}}^{-1}=Q_{x-z}^{-1}+Q_{y-z}^{-1}$$

This approximation assumes separation of Maxwell equations in the *x* and *y* directions and duplicates vertical optical losses, which may overestimate the three-dimensional optical losses. Nevertheless, in the case of a configuration with a square-shaped grating in the *x–y* plane, according to (2) optical losses in the *y* direction are responsible for a reduction in the *Q*-factor of approximately 10% compared to the value calculated for the same configuration in the *x–z* plane.

Inspection of the optical field distribution in the *x*–*z* plane indicates that the main channels of optical emission are through the top and bottom surfaces of the device. In the infinite grating configuration, the lateral components of the wavevectors are related to the first and second diffraction orders. In the finite structure, the distribution of the wavevector lateral components is not discrete [31]. Therefore, the wavevectors with transversal components smaller than those related to the first diffraction order are partially transmitted through the DBR, as are the wavevectors with transversal components larger than those of the second diffraction order. The transmission of these wavevectors with smaller and larger lateral components reduces the *Q*-factor of the studied configuration. The selective reflectivity of the DBR is a clear limitation compared with membrane configurations [31] or gratings implemented on low refractive index substrates [32], in which total internal reflection prohibits the propagation of wavevectors with large transversal components of the wavevectors in the substrate. Therefore, the finite configuration analyzed here may have a lower *Q*-factor compared to a membrane or grating implemented on a low refractive index substrate composed of a comparable number of stripes.

## 6 Conclusions

We have demonstrated the formation of an extended BIC in a system with vertically broken symmetry. By judiciously tuning the DBR to reflect higher diffraction orders produced by the grating, light propagating towards the DBR can be prevented from leaking. Such a system enables the creation of a BIC that is symmetry protected from the air side and protected by DBR reflection from the opposite side. It also enables a very high degree of design freedom with respect to the grating and DBR materials. *Q-*factors above 1000 can be achieved for structures composed of as few as six grating stripes, enabling small mode volumes. Increasing the number of grating stripes increases the *Q*-factor nearly exponentially. The *Q*-factors above 3000 achieved by the structures composed of more than 30 stripes facilitates stimulated emission [25]. This enables the fabrication of small dimensional lasers, which can be fabricated using conventional semiconductor technology—as shown by the example presented in Supplementary materials. Such an all-semiconductor configuration enables electrical injection, which is a significant step forward in the development of BIC-based lasers. Further reduction of the lateral size of the grating can be achieved by judicious structurization of the grating reducing mode volume [33].

To date, BICs have been theoretically predicted and experimentally demonstrated in periodic structures with high refractive index contrast between the periodic structure and surroundings such as thin membranes suspended in air [34], or implemented on a low refractive index substrate and then covered with a material that has an identical or almost identical refractive index to the substrate [5]. In the configuration demonstrated in this work, the effective refractive index of the periodic structure is lower than the refractive indices of the materials composing the DBR on which the periodic structure is implemented and the periodic structure is not covered by any material. Such a configuration is fundamentally different from any configuration in which BICs have been observed in the past. The periodic structure is implemented on a properly designed DBR and substrate, in a standard configuration for mature semiconductor epitaxy technology. Therefore, the configuration demonstrated in this paper significantly reduces the requirements for fabricating BIC cavities, and opens the way for more general applications of photonic resonators utilizing BICs.

## Supplementary Material

Supplementary Material
