# Supplementary material for: Extended bound states in the continuum in a one-dimensional grating implemented on a distributed Bragg reflector
Source: Nanophotonics. 2021 Nov 23;11(1):45–52. doi: 10.1515/nanoph-2021-0478 (PMC11501903; doi:10.1515/nanoph-2021-0478)
Supplement: Supplementary file 1 — Supplementary Material [file j_nanoph-2021-0478_suppl_001.pdf]

Supplementary Information

Extended bound states in the continuum in vertically nonsymmetric one-dimensional grating implemented on distributed Bragg reflector

Emilia Pruszyńska-Karbownik, Mikołaj Janczak, Tomasz Czyszanowski*

Lodz University of Technology, Institute of Physics, Photonics Group, Łódź, Poland

This PDF file includes:

Supporting information S1 and S2
Fig. S1, S2
Tab. S1

Supplementary S1: Numerical optical model

The optical model is based on the Plane Wave Admittance Method (PWAM) [S1]. The main objective of the method is the transformation of Maxwell's equations to a form of the characteristic equation. To this aim, the Cartesian coordinate system is oriented in such a way that the $x-y$ plane is parallel to the epitaxial layers, whereas the z direction is perpendicular to this plane. Then, the Maxwell's equations can be expressed in the form:

$$\nabla\times\mathbf{E}(x,y,z,t)=-\mu\mu_0\frac{\partial\mathbf{H}(x,y,z,t)}{\partial t} \qquad (1)$$

$$\nabla\times\mathbf{H}(x,y,z,t)=\varepsilon\varepsilon_0\frac{\partial\mathbf{E}(x,y,z,t)}{\partial t} \qquad (2)$$

with μ, μ_0 being the magnetic permittivity diagonal tensors for the material and vacuum, respectively, and ε, ε_0 being the respective diagonal tensors of dielectric constant. **E** and **H** are the vectors of the electric and magnetic fields. We further assume that:

1) the harmonic time dependence of the fields is of the form ~ exp($i\omega t$), with ω being the angular frequency of the wave.

2) the structure consists of uniform (in the propagation z-direction), parallel layers which yields Maxwell's set of the equations in the static form:

$$\nabla\times\mathbf{E}(x,y,z)=-i\omega\mu\mu_0\mathbf{H}(x,y,z) \qquad (3)$$

$$\nabla\times\mathbf{H}(x,y,z)=i\omega\varepsilon\varepsilon_0\mathbf{E}(x,y,z) \qquad (4)$$

Eliminating the z-components of the electric and magnetic fields from the above equations results in:

$$\partial_z^2\begin{bmatrix}E_x\\E_y\end{bmatrix}=\frac{1}{\omega^2\varepsilon_0\mu_0}\begin{bmatrix}-\partial_x\frac{1}{\varepsilon_z}\partial_x-\omega^2\varepsilon_0\mu_y\mu_0 & \partial_x\frac{1}{\varepsilon_z}\partial_y\\ -\partial_y\frac{1}{\varepsilon_z}\partial_x & \partial_y\frac{1}{\varepsilon_z}\partial_y+\omega^2\varepsilon_0\mu_x\mu_0\end{bmatrix}\times$$
$$\times\begin{bmatrix}-\partial_y\frac{1}{\mu_z}\partial_y-\omega^2\mu_0\varepsilon_x\varepsilon_0 & \partial_y\frac{1}{\mu_z}\partial_x\\ -\partial_x\frac{1}{\mu_z}\partial_y & \partial_x\frac{1}{\mu_z}\partial_x+\omega^2\mu_0\varepsilon_y\varepsilon_0\end{bmatrix}\begin{bmatrix}E_x\\E_y\end{bmatrix} \qquad (5)$$

The electromagnetic fields as well as the magnetic and electrical permittivities are decomposed in orthonormal, complete basis of exponential functions:

$$\Phi_u=\sum_{n,m}^{N}\tilde{\Phi}_u^{n,m}\varphi_{n,m} \qquad (6)$$

$$\eta_u=\sum_{n,m}^{\infty}\tilde{\eta}_u^{n,m}\varphi_{n,m} \qquad (7)$$

where Φ_u are the arbitrary field components of the electric or magnetic field, η_u are the components of magnetic or electric permittivity, and u = x, y, z. The basis functions have been

defined in the form of a product of two functions, which satisfy the othonormality and completeness of the basis.

$$\varphi_{n,m} = \exp\left(i\left(\frac{2\pi n}{L_x} + k_x \right) x + i\left(\frac{2\pi m}{L_y} + k_y \right) y \right), \qquad (8)$$

where L_x and L_y correspond to the dimensions of the calculation window along the x and y axis and k_x and k_y are corresponding components of the wavevector in the $x - y$ plane. Using these assumptions, the set of equations (5) is modified to the form in which fields and permittivities are replaced with coefficients of exponential expansions:

$$\partial_z^2 \begin{bmatrix} \tilde{E}_y^n \\ \tilde{E}_x^n \end{bmatrix} = -\frac{1}{k_0^2} \begin{bmatrix} (\mathbf{G}+\mathbf{k})(\mathbf{G}''+\mathbf{k})\tilde{\kappa}_z^{\mathbf{G}''-\mathbf{G}} - k_0^2 \tilde{\mu}_x^{\mathbf{G}''-\mathbf{G}} & -(\mathbf{G}+\mathbf{k})(\mathbf{G}''+\mathbf{k})\tilde{\kappa}_z^{\mathbf{G}''-\mathbf{G}} \\ (\mathbf{G}+\mathbf{k})(\mathbf{G}''+\mathbf{k})\tilde{\kappa}_z^{\mathbf{G}''-\mathbf{G}} & k_0^2 \tilde{\mu}_y^{\mathbf{G}''-\mathbf{G}} - (\mathbf{G}+\mathbf{k})(\mathbf{G}''+\mathbf{k})\tilde{\kappa}_z^{\mathbf{G}''-\mathbf{G}} \end{bmatrix} \times$$
$$\times \begin{bmatrix} (\mathbf{G}+\mathbf{k})(\mathbf{G}''+\mathbf{k})\tilde{\gamma}_z^{\mathbf{G}''-\mathbf{G}} - k_0^2 \tilde{\varepsilon}_y^{\mathbf{G}''-\mathbf{G}} & -(\mathbf{G}+\mathbf{k})(\mathbf{G}''+\mathbf{k})\tilde{\gamma}_z^{\mathbf{G}''-\mathbf{G}} \\ (\mathbf{G}+\mathbf{k})(\mathbf{G}''+\mathbf{k})\tilde{\gamma}_z^{\mathbf{G}''-\mathbf{G}} & k_0^2 \tilde{\varepsilon}_x^{\mathbf{G}''-\mathbf{G}} - (\mathbf{G}+\mathbf{k})(\mathbf{G}''+\mathbf{k})\tilde{\gamma}_z^{\mathbf{G}''-\mathbf{G}} \end{bmatrix} \begin{bmatrix} \tilde{E}_y^{\mathbf{G}} \\ \tilde{E}_x^{\mathbf{G}} \end{bmatrix} \qquad (9)$$

The boundary conditions assumed for the set of equations are fulfilled by absorbing Perfectly Matched Layers [S2] in the case of finite structures. For simulation of structures that are infinite in the *x*-*y* directions we simulate a single period of the structure with periodic boundary conditions. The final set (9) can be solved in a base, which reduces the product of matrices from (9) to diagonal forms:

$$\partial_z^2 \hat{\mathbf{E}} + \mathbf{\Gamma}_{\mathbf{E}}^2 \hat{\mathbf{E}} = 0 \qquad (10)$$

In the same manner one arrives at the equation for the magnetic field:

$$\partial_z^2 \hat{\mathbf{H}} + \mathbf{\Gamma}_{\mathbf{H}}^2 \hat{\mathbf{H}} = 0, \qquad (11)$$

where $\hat{\mathbf{E}}$ and $\hat{\mathbf{H}}$ stand for the electric and magnetic fields in the new base. The solutions of equations (10) and (11) have the well known form of a standing wave:

$$\hat{\mathbf{E}}(z) = \cosh\left(i\mathbf{\Gamma}_{\mathbf{E}} z\right)\mathbf{A}_{\mathbf{E}} + \sinh\left(i\mathbf{\Gamma}_{\mathbf{E}} z\right)\mathbf{B}_{\mathbf{E}} \qquad (12)$$

$$\hat{\mathbf{H}}(z) = \cosh\left(i\mathbf{\Gamma}_{\mathbf{H}} z\right)\mathbf{A}_{\mathbf{H}} + \sinh\left(i\mathbf{\Gamma}_{\mathbf{H}} z\right)\mathbf{B}_{\mathbf{H}} \qquad (13)$$

The above relations of the field transformation between and within the layers can be employed to determine the characteristic equation by forcing boundary conditions, i.e. by a zeroing of the field on the upper-most and lower-most layer boundaries. This then allows us to find the characteristic values of the problem, i.e. the resonance wave number (k_0) of the microcavity, as well as the characteristic vectors, which determine the distribution of the electromagnetic field within the structure.

In the case of determining the power reflectance we solve the problem of a propagating wave and assume that in the last layer behind the mirror there is no in-going wave and the incident wave is a plane wave in the first layer in front of the mirror.

Concerning the reduction of the numerical error in the solution of the resonance problem one can choose a matching interface, which should be placed at a maximum of the optical field intensity standing wave. This leads to the following relationship between electric fields in neighboring layers:

$$\mathbf{T}_{\mathbf{H}}^{(m)}\mathbf{Y}_{up}^{(m)}\hat{\mathbf{E}}_{d}^{(m)} = \mathbf{T}_{\mathbf{H}}^{(m)}\mathbf{Y}_{down}^{(l)}\hat{\mathbf{E}}_{d}^{(l)} \tag{14}$$

where:

$$\mathbf{Y}^{(i)} = -\left(\mathbf{y}_{2}^{(i)}\left(\mathbf{t}_{\mathbf{H}}^{(i)}\mathbf{Y}^{(i-1)}\left(\mathbf{t}_{\mathbf{E}}^{(i)}\right)^{-1} - \mathbf{y}_{1}^{(i)}\right)^{-1}\mathbf{y}_{2}^{(i)} + \mathbf{y}_{1}^{(i)}\right), \tag{15}$$

i.e. the matrices $\mathbf{Y}$ are determined from a recurrence relation starting from the bottom $\mathbf{Y}_{up}^{(m)}$ and the top $\mathbf{Y}_{down}^{(m)}$ of the microcavity. Thus, Eq. (14) results in:

$$\left(\mathbf{T}_{\mathbf{H}}^{(m)}\mathbf{Y}_{up}^{(m)}\left(\mathbf{T}_{\mathbf{E}}^{(m)}\right)^{-1} - \mathbf{T}_{\mathbf{H}}^{(m)}\mathbf{Y}_{down}^{(l)}\left(\mathbf{T}_{\mathbf{E}}^{(l)}\right)^{-1}\right)\bar{\mathbf{E}} = \mathbf{Y}\bar{\mathbf{E}} = 0, \tag{16}$$

which is the characteristic value equation. The solution of Eq. (16) determines the complex value of k_0. Its imaginary part determines the modal gain of the mode.
The complex wavevector k_0 and corresponding resonant wavelength (λ) satisfy [S3]:

$$k_0^2 = (2\pi/\lambda)^2 = k_z^2 + k_y^2 \tag{17}$$

where k_z is the wavenumber component determined by the vertical geometry of the microcavity and k_y is the lateral component. The lateral component can be 0 if the resonating wave is a planewave and propagates along the z direction. This is the case for vertical resonance between two parallel mirrors that are infinite in their lateral direction. If the mirrors are finite in the y direction, the lateral size of the cavity mode is determined by the size of the mirrors and the lateral component of k_0 emerges. By reducing the lateral size of the cavity mode we observe a blue-shift of the resonant wavelength which relates to the increase in k_0 induced by the increase of k_y. The lateral components of k_0 contribute to the wave propagation out of the cavity's optical axis. From the complex value of k_0 we determine the quality (Q) factor of the cavity according to the formula [S4]:

$$Q = -\ 0.5\ k_{0re}/k_{0im}, \tag{18}$$

where k_{0re} is the real part of k_0 and k_{0im} is the imaginary part of k_0.
The simulated region of size W is determined by the lateral size of the MHCG together with additional zones of three wavelengths in width between the MHCG and the PML. Larger simulation areas are used to illustrate mode distributions in the cavity and in the cavity's surroundings to produce the results given in Fig. 6 of the main text. The number of plane waves used in the method was approximated according to the formula: $W/L\times30$, where L is the grating segment width. Due to our computational capabilities, the maximum number of grating stripes we modelled is limited to 100. To determine the power reflectance of the structure as well as k_0 we

simulate infinite structures with appropriate periodic boundary conditions as described earlier and we use 30 plane waves to reach convergence.

Supplementary S2: Arsenide-based configuration

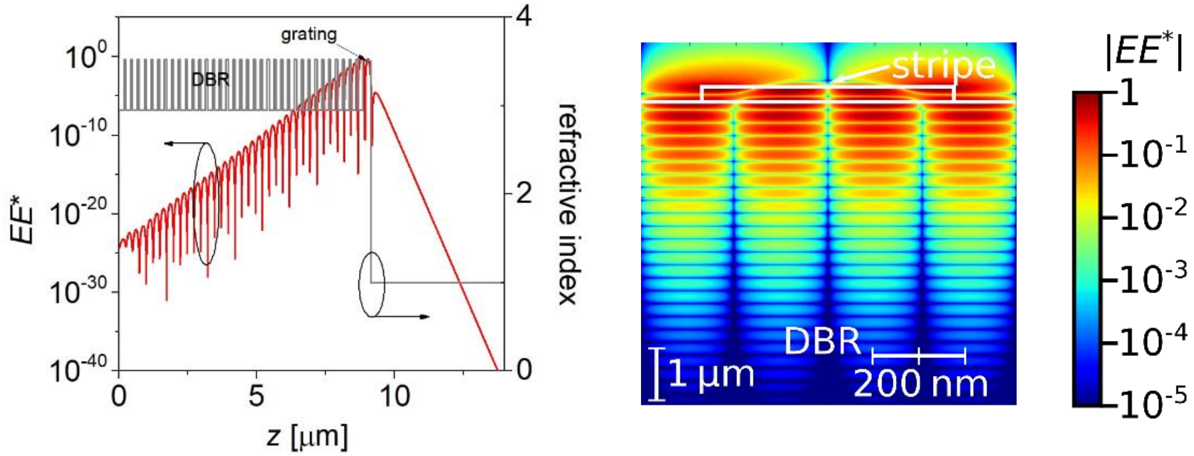

a) b)

Fig. S1 Light intensity profiles a) along the *z* axis in the cavity with arsenide DBR. The grey line represents the profile of the refractive index along the *z* axis; b) in *x*-*z* plane. Geometrical details of the configuration are collected in Tab. S1

Table S1 Geometrical details of the arsenide-based structure for unity wavelength.

Layer	Dimensions	Value	Refractive index
Grating	Height *H*	0.300	3.521
	Period *L*	0.849	
	Fill-factor *F*	0.675	
Low-refractive-index DBR layer	Thickness	0.183	2.951
High-refractive-index DBR layer	Thickness	0.074	3.521

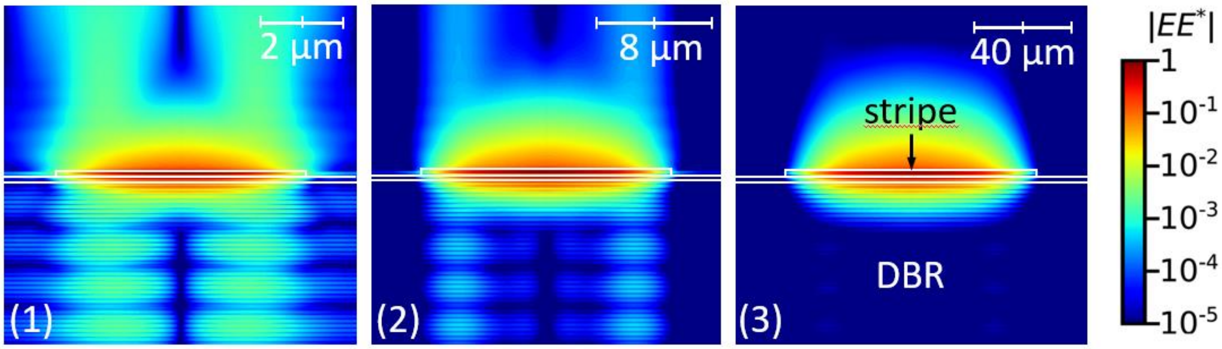

Fig. S2 Light intensity distributions in *y*-*z* plane on a logarithmic scale of colors for structures composed of grating stripes that lengths are approximately 6, 16, 80 μm. *x* position is shifted 0.1 μm from the center of the stripe where light intensity is 0 due to the mode symmetry.

Supplementary references

[S1] Dems M, Kotynski R, Panajotov K. Plane wave admittance method—A novel approach for determining the electromagnetic modes in photonic structures. Opt. Exp. 2005;13:3196–207.

[S2] Mittra R, Pekel U. New look at the perfectly matched layer (PML) concept for the reflectionless absorption of electromagnetic waves, IEEE Microw. Guid. Wave Lett. 1995;5:84–6.

[S3] Saleh BEA, Teich MC. Fundamentals of photonics, New Jersey: Wiley, 2002.

[S4] Karagodsky V, Pesala B, Chase C, Koyama F, Chang-Hasnain CJ. Monolithically integrated multi-wavelength VCSEL arrays using high-contrast gratings, Opt. Exp. 2010; 18: 694–9.
